# Supplementary material for: Learning curve and surgical outcome of robotic assisted colorectal surgery with ERAS program
Source: Sci Rep. 2022 Nov 29;12:20566. doi: 10.1038/s41598-022-24665-w (PMC9709162; doi:10.1038/s41598-022-24665-w)
Supplement: Supplementary file 2 — Supplementary Information 2. [file 41598_2022_24665_MOESM2_ESM.zip › RAL Raw data-Ñ[▒K/054/OP N_ñw╝╨░O▒Kñσ.pdf]

W52 / W52

臺中榮民總醫院
手術室護理記錄

[redacted]

基本資料

- 室別： 開刀房-17室，手術科別： GS+CRS，手術類別： 常規，日期： 2021/01/20
- 進入時間： 1600 ； 麻醉開始時間： 1605 ； Time Out時間： 1735 ； 劃刀時間： 1736 ； 手術結束時間： 2240 送出時間： 2250 ；
- 麻醉方式： GE

  診斷：LIVER CYST

  手術名稱：1.Robot Assisted Colectomy(L)
  2.Liver Laparoscopic fenestration for hepatic cyst

  手術局部用藥：NIL

  術前預防性抗生素：
  - 抗生素名稱一： Veterin 1GM IVD由麻醉護理師給予
  - 時間： 1715
  - 上次簽章: 馬景榆

  ，追加預防性抗生素：
- 手術醫師： 吳峰旭，林俊余，古朋育，林陳彥，林辰祐
- 麻醉醫師： VS 吳志成
-
  - 刷手護士A ： 羅媗鈴，時間： 1600，刷手護士B ： 廖子晴，時間： 1600，接替刷手一： 陳慧瑜，時間： 1900
  - 流動護士A ： 馬景榆，時間： 1600，接替流動一： 陳慧瑜，時間： 1800，接替流動二： 馬景榆，時間： 1900
- 更多接替及流動護士： 接替流動三： 鍾佩方，時間： 2000

皮膚狀況

A-V Shunt： 無

術前： 完整

記錄者： 上次簽章: 馬景榆

術後： 完整

記錄者： 鍾佩方

手術準備

手術臥式 單一手術臥式

-
  - 手術臥式： 截石臥式
  - 使用附件： 溫毯 38 ℃，頭圈，腳架，其它： silicon rod*2、lateral support
  - 皮膚準備： 10%Aq Betadine，Hibiscrub，2% Chlorhexidine
  - 電燒： 使用電燒: 電極片位置： 左大腿，右大腿，記錄者： 上次簽章: 馬景榆

止血帶T： 無

植入物

- 植入物名稱/號碼/數量/部位：Stapler GIA/45mm-4.3mmX2/S-colon

，記錄者： 鍾佩方

標本送檢

- 細菌培養 一般，採取部位: 1. cyst，2. cyst，數量： 2
- 細胞學 無
- 冰凍切片 無
- 病理標本

  組織名稱一：cyst

  ,

  組織名稱二：S-colon

  ,

組織名稱三： IMA ROOT
，更多組織名稱

- 組織名稱四： Distal cut end

- 截肢 無

標本交付 標本： 2 袋 ，標本： 0 盒 ，標本： 2 瓶 ，細胞學： 0 管 ，Culture Tube： 2 管 ，被交付醫師： CR 林陳彥，

- 記錄者： 鍾佩方
- 時間： 2250

傷口術後數料

傷口數料： 傷口數料： 無

- 請於圖上標示部位：

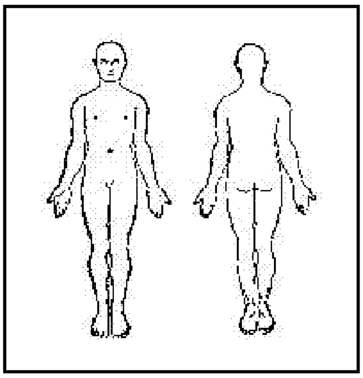

- 傷口數料： mepore+紗布

導管

導管	天數	導管資訊	插入日期	插入時間	更換日期	拔除日期	拔除時間	拔除原因	預計拔除/更換	預計拔除/更換日期
Epidural PCA(1)	1	部位:硬脊膜;	2021/01/20	1610						
Foley(1)	1	號碼:16 Fr.; 部位:膀胱;	2021/01/20	1700						
Sump NG Tube(1)	1	號碼:16 Fr.; 深度:60cm; 部位:胃;	2021/01/20	1700						
Double Lumen CVC(1)	1	號碼:8 Fr.; 深度:15cm; 部位:jugular (右); 象限:RU;	2021/01/20	1650						
A-line (1)	1	號碼:20 G; 部位:左手;	2021/01/20	1650						

用物計數

- 手術中出血量： 少 c.c.
- 手術中尿量： 800 c.c.
-
  - 其它： 有
  - cyst:1450ml

用物計數： 多切口同時縫合

- 1.器械： 正確，2.縫針： 正確，3.刀片： 正確，4.紗布： 正確，5.腹部墊： 未使用，6.紗球： 未使用，7.PEANUTS ： 未使用，8.紗條： 未使用，9.絲帶： 未使用，10.針頭： 未使用，11.Vessel loop： 未使用，12.Boots： 未使用，13.Jelco： 未使用，14.棉條： 未使用，
  - 第一次：
    - 刷手護士 ： 陳慧瑜
    - 流動護士： 鍾佩方
    - 時間： 2200

  ，
  - 第二次：
    - 刷手護士 ： 陳慧瑜
    - 流動護士： 鍾佩方
    - 時間： 2210

,

- 第三次：
  - 刷手護士 ： 陳慧瑜
  - 流動護士： 鍾佩方
  - 時間： 2220

備註

備註： NIL

- 記錄者： 上次簽章: 馬景榆
- 時間： 2000

填寫護理人員 鍾佩方

正式摘要

W52 / W52

doctorRank:B
